# Highly Efficient and Enzymatic Regioselective Undecylenoylation of Gastrodin in 2-Methyltetrahydrofuran-Containing Systems

**DOI:** 10.1371/journal.pone.0110342

**Published:** 2014-10-16

**Authors:** Rongling Yang, Xueming Liu, Zhiyi Chen, Chunying Yang, Yaosheng Lin, Siyuan Wang

**Affiliations:** Sericulture and Agri-Food Research Institute, Guangdong Academy of Agricultural Sciences, Guangzhou, China; Instituto de Tecnologica Química e Biológica, UNL, Portugal

## Abstract

Highly efficient and regioselective acylation of pharmacologically interesting gastrodin with vinyl undecylenic acid has been firstly performed through an enzymatic approach. The highest catalytic activity and regioselectivity towards the acylation of 7′-hydroxyl of gastrodin was obtained with *Pseudomonas cepacia* lipase. In addition, it was observed the lipase displayed higher activity in the eco-friendly solvent 2-methyltetrahydrofuran-containing systems than in other organic solvents. In the co-solvent mixture of tetrahydrofuran and 2-methyltetrahydrofuran (3/1, v/v), the reaction rate was 60.6 mM/h, substrate conversion exceeded 99%, and 7′-regioselectivity was 93%. It was also interesting that the lipase-catalyzed acylation couldn’t be influenced by the benzylic alcohol in gastrodin. However, *pseudomonas cepacia* lipase displayed different regioselectivity towards gastrodin and arbutin.

## Introduction

**Figure 1:**
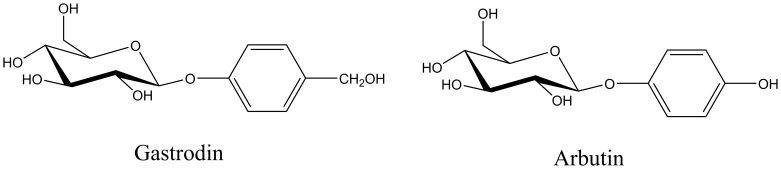
Chemical construction of gastrodin and arbutin.

Gastrodin, namely *p*-hydroxymethy-lphenyl-*β*-D-glucopyranoside (Figure 1), is one of the major active ingredient obtained from *Gastrodia elata* Blume with extensive pharmacological activities. It has been clinically used due to its sedative, hypnotic, anticonvulsion and neuroprotective effect, while no side effects and toxicities in patients have been observed so far [1], [2]. However, gastrodin usually suffers from low oral bioavailability because it is difficult to penetrate cell membrane. It has been demonstrated that glucoside ester compounds could be good glyco-drugs [3] and improve bioavailablity of some drugs [4]. So, gastrodin bioavailability might be enhanced significantly by acylation with the fatty acid. Recently, it was reported that some gastrodin analogues bearing lipophilic groups in glycosyl moiety had higher anti-influenza activities [5], presumably due to their improved membrane penetration.

Gastrodin is a polyhydroxy compound, which possesses several hydroxyls with similar chemical activity. Generally, it is very hard to selectively acylate the desired hydroxyl through the classical chemical methods unless gastrodin was protected. Fortunately, the enzymatic regioselective acylation of polyhydroxy compounds serves as a useful alternative to traditional chemical approach, owing to the excellent regioselectivity, simplicity and environmental friendliness [6], [7], [8], [9], [10]. We previously investigated the enzymatic regioselective acylation of arbutin (Figure 1), whose chemical structure is same as gastrodin except for benzylic alcohol [11]. So, it was also interesting whether the enzymatic activity and selectivit may be influenced by the benzylic alcohol in gastrodin.

Recently, the environment-friendly solvent 2-methyltetrahydrofuran has been increasingly applied in biotransformation due to its environmental friendly and renewable characteristics [12], [13], [14], [15]. For example, in the regioselective acylation of cordycepin and purine nucleoside analogs, immobilized lipases from *Candida antarctica* and *Penicillium expansum* showed higher catalytic activity and preferable thermostability in MeTHF [12], [13]. To our knowledge, the enzymatic acylation of gastrodin in MeTHF-containing systems have never been reported.

**Figure 2:**
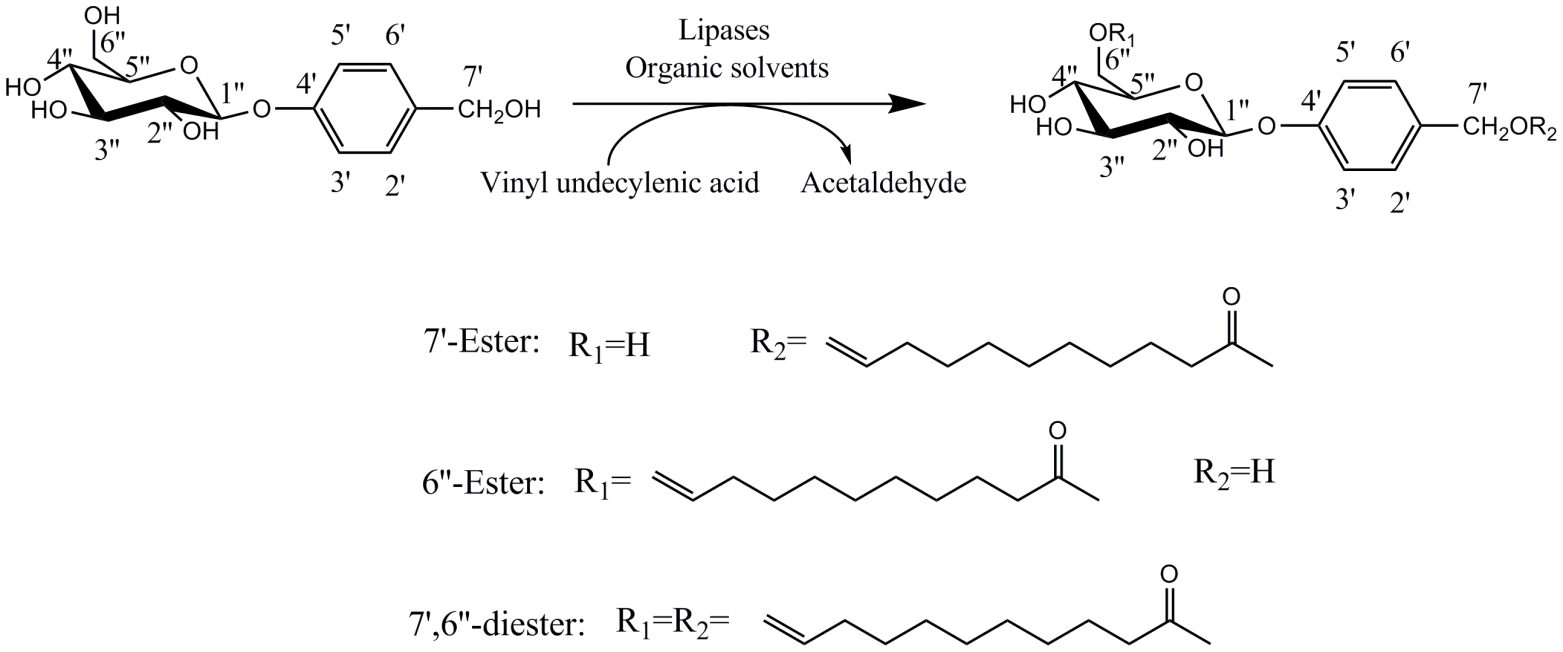
Enzymatic regioselective acylation of gastrodin with vinyl undecylenic acid.

Undecylenic acid (11∶1, cis-10) possesses a C–C double bond, which was found in the body (occurring in sweat). Li reported that undecylenic acid glucoside esters could be used as initial materials to synthesize functional polymeric prodrugs with controlled release [16]. In addition, it is a novel non-peptide-like µ-calpain inhibitor with good cell permeability and possesses antifungal, antiviral, insect-repelling and potent neuroprotective activities [17], [18]. Encouraged by this, the enzymatic regioselective acylation of gastrodin with vinyl undecylenic acid was firstly carried out (Figure 2), for the production of novel gastrodin derivatives with potential pharmacological activities.

## Materials and Methods

### Biological and chemical materials

Lipase B from *Candida antarctica* (CAL-B), lipase from Thermomyces lanuginosus (TL IM) and lipase from *Rhizomucor miehei* (Lipozyme RML) are immobilized lipases from Novozymes Co., Ltd., China. The immobilized lipase from *Burkholderia cepacia* (PS IM) was purchased from Amano Enzyme Inc., Japan. Lipase powder from *Candida rugosa* (CRL) was from Meito SangyoCo., Japan. Lipase from *Penicillium roqueforti* (PRL) and lipase from *Penicillium camemberti* (PCL) are powders from Amano Enzyme Inc., Japan. Gastrodin and undecylenic acid vinyl ester were from TCI, Japan. The solvents were dried with 4 Å molecular sieves with gentle shaking overnight prior to use. Other chemicals were from commercial sources and were of the highest purity available. And the boiling range of petroleum ether used in esters purification was 60–90°C.

### Determination of lipase activity

The lipase activity was was assayed with *p*-nitrophenyl acetate hydrolysis as a model reaction [19]. In a typical experiment, 0.6 mL phosphate buffer (50 mM, pH 8.0) containing 0.1 mL *p*-nitrophenyl acetate (10 mM dissolve in 2-propanol) and the enzyme was incubated at 45°C and 200 rpm for 20 min, then stopped by adding 0.1 M Na_2_CO_3_ solution. After centrifugation, the supernatant was measured at 410 nm by spectrophotometer. One unit of lipase activity was defined as the amount of enzyme required to produce 1 µmol *p*-nitrophenol per min under the above conditions. The specific activities of CAL-B, TLL, PS IM, RML, CRL, PCL and PRL were 2.42, 0.20, 0.57, 0.25, 0.63, 0.10 and 2.63 U/mg, respectively.

### Enzymatic acylation of gastrodin

In a typical experiment, 2 mL anhydrous organic solvent containing 0.04 mmol gastrodin, 15 U lipases and 0.2 mmol undecylenic acid vinyl ester were incubated at 45°C and 200 rpm. Aliquots were withdrawn at specified time intervals from the reaction mixture, and diluted 25 times with methanol prior to HPLC analysis. All reported data were averages of experiments performed in triplicate. The controls were performed by following the above procedure except that no enzyme was added, and no chemical acylation of arbutin was detectable. Regioselectivity was defined as the ratio of the HPLC peak area corresponding to the desired product to that of all the products formed. The initial reaction rate (V_0_) and the substrate conversion (*conv*.) were calculated from the HPLC data according to the following equations:
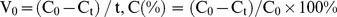
where *C_0_* and *C_t_* represent the initial substrate concentration and the substrate concentration after reaction for a certain time (*t*), respectively, and *t* stands for the reaction time.

### Operational stability

In this study, 2 mL anhydrous THF-MeTHF (3/1, v/v) containing 0.04 mmol gastrodin, 0.20 mmol vinyl undecylenic acid and 15 U lipase PS IM was incubated at 200 rpm and 45°C for 0.5 h. The lipase was filtered and thoroughly washed with THF-Me THF (3/1, v/v). Next, the washed lipase was added into fresh reaction mixture with 0.04 mmol gastrodin and 0.20 mmol vinyl undecylenic acid.

### HPLC analysis

The analysis was performed using the Agilent HPLC system (Agilent Technologies Industries Co., Ltd., USA) consisting of a G1311A pump and a UV detector. The reaction mixture was analyzed by RP-HPLC on a Zorbax SB-C18 column (250 mm×4.6 mm, 5 µm, Agilent Technologies Industries Co., Ltd., USA). The wavelength of the UV detector was set at 220 nm with a flow rate of 1.0 mL/min and column temperature at 25°C.

A gradient mobile phase system was water and methanol. The gradient elution method was as follows: 0–8 min: 80% methanol; 8–12 min: 80% methanol→90% methanol; 12–20 min: 90% methanol; 20–21 min: 90% methanol→100% methanol; 21–30 min: 100% methanol. The retention times for gastrodin, 6 ″–, 7′-undecylenic acid and 7′,6″-diundecylenic acid of gastrodin were 2.39, 5.55, 8.2 and 24.38 min, respectively.

### Purification and structure determination of gastrodin esters

Upon the completion of the reaction, the lipase PS IM was filtered off and the solvent was concentrated under vacuum. The residue was then purified by flash column chromatography using ethyl acetate/petroleum ether (boiling poin: 60–90°C) as an eluant. The structures of gastrodin ester derivatives were determined by NMR (Bruker AVANCE Digital 400 MHz NMR spectrometer, Germany) and mass spectra (Bruker maXis impact, Bruker Co., Germany) using ESI mode. The position of acylation in enzymatically prepared ester was determined by ^13^C NMR (100 MHz) and ^1^H NMR (400 MHz).

### Gastrodin

*m/z*: 309.0950 [M+Na]^+^. C_13_H_18_O_7_.

^13^C NMR (DMSO-*d*_6_): *δ* 60.70 (C_6″_), 62.48 (C_7′_), 69.72 (C_4″_), 73.24 (C_2″_), 76.62 (C_5″_), 76.97 (C_3″_), 100.54 (C_1″_), 115.92 (C_3′_+ C_5′_), 127.66 (C_2′_+ C_6′_), 135.82 (C_1′_), 156.29 (C_4′_). ^1^H NMR (DMSO-*d*_6_): *δ* 3.47 (d, 1H, *J* = 5.6 Hz, H_6″_), 3.69 (d, 1H, *J* = 10.4 Hz, H_6″_), 4.41 (s, 2H, H_7′_), 4.82 (d, 1H, *J* = 7.6 Hz, H_1″_), 6.98 (d, 2H, *J* = 8.4 Hz, H_3_+ H_5_), 7.22 (d, 2H, *J* = 8.4 Hz, H_2_+ H_6_).

### Gastrodin 7′-undecenoate

*m/z*: 475.2304 [M+Na]^+^. C_24_H_36_O_8_.

^13^C NMR (DMSO-*d*_6_): *δ* 24.91 (C_3_), 28.69 (C_7_), 28.87 (C_6_+ C_5_), 29.07 (C_4_), 29.14 (C_8_), 33.63 (C_2_), 33.95 (C_9_), 61.16 (C_6″_), 65.50 (C_7′_), 70.17 (C_4″_), 73.68 (C_2″_), 77.08 (C_5″_), 77.51 (C_3″_), 100.81 (C_1″_), 115.10 (C_11_), 116.61 (C_3′_+ C_5′_), 129.99 (C_1′_), 130.08 (C_2′_+ C_6′_), 139.29 (C_10_), 157.70 (C_4′_), 173.27 (C_1_). ^1^H NMR (DMSO-*d*_6_): *δ* 1.23 (s, 8H, H_7_+ H_5_+ H_6_+ H_8_), 1.29–1.38 (m, 2H, H_4_), 1.44–1.55 (m, 2H, H_3_), 2.02 (q, 2H, *J* = 13.6 Hz, H_9_), 2.32 (t, 2H, *J* = 7.2 Hz, H_2_), 3.13–3.33 (m, 5H, H_2″_+ H_3″_+ H_4″_+ H_5″_+ OH_6″_), 3.47 (d, 1H, *J* = 6.0 Hz, H_6″_), 3.69 (d, 1H, *J* = 10.8 Hz, H_6″_), 4.54 (s, 1H, OH_4v_), 4.86 (d, 1H, *J* = 7.2 Hz, H_1″_), 4.97 (apparent t, 2H, *J* = 11.6 Hz, H_11_), 5.00 (s, 2H, H_7′_+ OH_3″_), 5.31 (s, 1H, OH_2″_), 5.73–5.83 (m, 1H, H_10_), 7.01 (d, 2H, *J* = 8.4 Hz, H_3′_+ H_5′_), 7.29 (d, 2H, *J* = 8.4 Hz, H_2′_+ H_6′_).

### Gastrodin 6″-undecenoate

*m/z*: 475.2306 [M+Na]^+^. C_24_H_36_O_8._

^13^C NMR (DMSO-*d*_6_): *δ* 24.88 (C_3_), 28.72 (C_7_), 28.93 (C_6_+ C_5_), 28.99 (C_4_), 29.16 (C_8_), 33.63 (C_2_), 33.99 (C_9_), 62.95 (C_6″_), 63.83 (C_7′_), 70.45 (C_4″_), 73.63 (C_2″_), 74.17 (C_5″_), 76.81 (C_3″_), 100.81 (C_1″_), 115.07 (C_11_), 116.33 (C_3′_+ C_5′_), 128.04 (C_2′_+ C_6′_), 136.44 (C_1′_), 139.28 (C_10_), 156.58 (C_4′_), 173.18 (C_1_). ^1^H NMR (DMSO-*d*_6_): *δ* 1.22 (s, 8H, H_7_+ H_5_+ H_6_+ H_8_), 1.31 (apparent s, 2H, H_4_), 1.49 (apparent d, 2H, *J* = 6.8 Hz, H_3_), 2.00 (d, 2H, *J* = 6.8 Hz, H_9_), 2.29 (t, 2H, *J* = 7.2 Hz, H_2_), 3.12–3.28 (m, 3H, H_2″_+ H_3″_+ H_4″_), 3.59 (t, 1H, *J* = 8.0 Hz, H_5″_), 4.07 (dd, 1H, *J* = 7.2, 12 Hz, H_6″_), 4.33 (d, 1H, *J* = 10.4 Hz, H_6″_), 4.42 (d, 2H, *J* = 5.2 Hz, H_7′_), 4.85 (d, 1H, *J* = 7.6 Hz, H_1″_), 4.96 (t, 2H, *J* = 10.8 Hz, H_11_), 5.08 (t, 1H, *J* = 6.0 Hz, OH_7′_), 5.20 (d, 1H, *J* = 3.2 Hz, OH_4″_), 5.28 (d, 1H, *J* = 5.2 Hz, OH_3″_), 5.38 (d, 1H, *J* = 4.8 Hz, OH_2v_), 5.72–5.83 (m, 1H, H_10_), 6.95 (d, 2H, *J* = 8.8 Hz, H_3′_+ H_5′_), 7.22 (d, 2H, *J* = 8.8 Hz, H_2′_+ H_6′_).

### Gastrodin 7′, 6″-undecenoate

*m/z*: 641.3673 [M+Na]^+^. C_35_H_54_O_9._

^13^C NMR (DMSO-*d*_6_): *δ* 24.87 (C_3b_), 24.91 (C_3s_), 28.70 (C_7b_), 28.73 (C_7s_), 28.89 (C_5s_+ C_5b_), 28.93 (C_6s_+ C_6b_), 29.07 (C_4s_+ C_4b_), 29.14 (C_8b_), 29.18 (C_8s_), 33.63 (C_2s_+ C_2b_), 33.97 (C_9s+_C_9b_), 63.85 (C_6′'_), 65.43 (C_7′_), 70.48 (C_4′'_), 73.56 (C_2′'_), 74.20 (C_5′'_), 76.79 (C_3′'_), 100.52 (C_1′'_), 115.05 (C_11s_+ C_11b_), 116.54 (C_3′_+ C_5′_), 129.96 (C_2′_+ C_6′_), 130.10 (C_1′_), 139.26 (C_10s_+ C_10b_), 157.48 (C_4′_), 173.13 (C_1b_), 173.18 (C_1s_). ^1^H NMR (DMSO-*d*_6_): *δ* 1.22 (s, 16H, H_7(b,s)_+ H_5(b,s)_+ H_6(b,s)_+ H_8(b,s)_), 1.30 (apparent s, 4H, H_4(b,s)_), 1.50 (d, 4H, *J* = 7.2 Hz, H_3(b,s)_), 1.99 (apparent d, 4H, *J* = 4.0 Hz, H_9(b,s)_), 2.24–2.31 (m, 4H, *J* = 7.2 Hz, H_2(b,s)_), 3.16 (t, 1H, *J* = 9.2 Hz, H_2′'_), 3.22–3.3v3 (m, 3H, H_3″_+ H_4″_+ H_5″_), 3.61 (t, 1H, *J* = 7.6 Hz, H_11(b/s)_), 4.06 (dd, 1H, *J* = 7.2, 11.6 Hz, H_6″_), 4.32 (d, 1H, *J* = 10.4 Hz, H_6″_), 4.89 (d, 1H, *J* = 7.2 Hz, H_1″_), 4.95 (t, 3H, *J* = 10.0 Hz, H_11(b,s)_), 5.00 (s, 3H, H_7′_+ OH_4″_), 5.30–5.36 (m, 2H, OH_2″_+ OH_3″_), 5.72–5.82 (m, 2H, H_10(b,s)_), 6.99 (d, 2H, *J* = 8.4 Hz, H_3′_+ H_5′_), 7.27 (d, 2H, *J* = 8.4 Hz, H_2′_+ H_6′_).

## Results and Discussion

### Regioselectivity acylation of gastrodin

Lipases are the most versatile biological catalyst in the synthesis proces of pharmaceutical, food and cosmetic sectors because of their catalytic versatility, excellent selectivity and environmental friendliness [20], [21]. Therefore, four immobilized lipases (CAL-B, TLL, PS IM and RML) and three enzyme powders (PCL, PRL and CRL) were screened for their capacity in the regioselective acylation of gastrodin with vinyl undecylenic acid (Table 1). Among these lipases, lipozyme PS IM and TLL showed the high catalytic activities (40.3 and 32.2 mM/h, respectively), while lipases CAL-B and RML displayed low reaction rate and low conversion. In addition, three enzyme powders (PCL, PRL and CRL) showed no catalytic activity in the reaction.

**Table 1:** Regioselective acylation of gastrodin with vinyl undecylenic acid catalyzed by various lipases.

Enzyme	V_0_ (mM/h)	Time (h)^a^	*C* (%)	7'-Ester (%)	6″-Ester (%)	7',6″-diester (%)
CAL-B	17.2	8	76	86	2	12
TLL	32.2	2.5	92	17	15	68
PS IM	40.3	2	98	95	1	4
RML	6.6	10	42	99	0	0
PCL	n.d.	48	n.d.	n.d.	n.d.	n.d.
PRL	n.d.	48	n.d.	n.d.	n.d.	n.d.
CRL	n.d.	48	n.d.	n.d.	n.d.	n.d.

Reaction conditions: 20 mM gastrodin, 100 mM vinyl undecylenic acid, 15 U lipases, 2 mL anhydrous THF, 45°C, 200 rpm.

a Reaction time when the maximum conversion was achieved.

n.d.: no detected.

Interestingly, the lipases PS IM, CAL-B and RML displayed excellent regioselectivities towards primary hydroxyl group at benzylic alcohol in the acylation, while lipase TLL was favorable for the diacylated derivatives synthesis of parent compound. However, the previous study found that the lipases PS IM, CAL-B and TLL displayed absolute regioselectivities (>99%) towards primary hydroxyl at glucose moiety in arbutin acylation [11], whose chemical structure is same as gastrodin except for benzylic alcohol. Likewise, during acylation of flavonoid glycosides and saccharides, lipase PS, CAL-B and TLL, exhibited an excellent selectivity for primary hydroxyl at the glucose moiety [22], [23], [24], [25], [26]. These results suggest that 7′-OH at benzylic alcohol of gastrodin have an easier access to the active site of lipases to attack the acyl-enzyme intermediate than 6″-OH of the sugar moiety, perhaps due to less steric hindrance factor, thus resulting in preferential acylation at the 7′-OH at benzylic alcohol.

### Time course of enzymatic reaction

**Figure 3:**
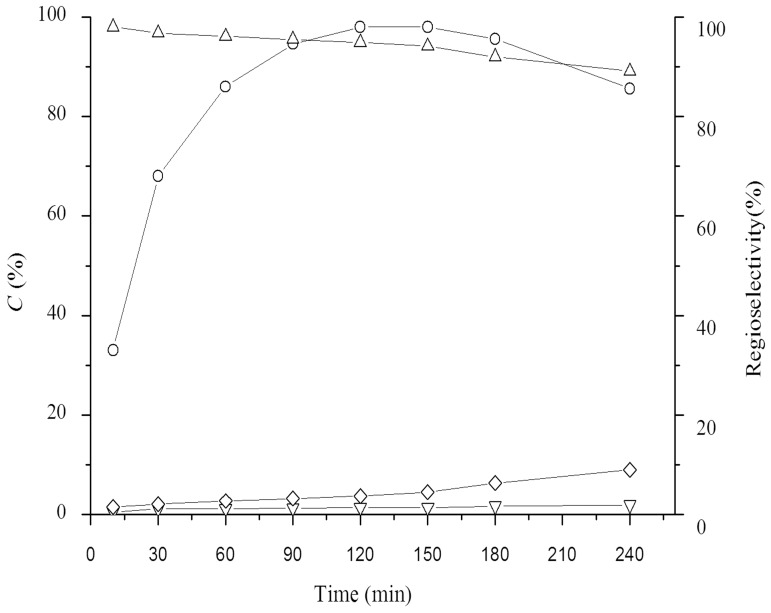
Time course of enzymatic undecylenoylation of gastrodin catalyzed by lipase PS IM. Reactions conditions: 20 mM gastrodin, 100 mM vinyl undecylenic acid, 15 U lipase PS IM, 2 mL anhydrous THF, 45°C, 200 rpm. Symbols: (○) conversion, (△) 7′-regioselectivity, (▽) 6″-regioselectivity, (◊) 7′,6″-regioselectivity.

To further understand the enzymatic acylation process, the reaction process of undecylenoylation of gastrodin catalyzed by lipase PS IM was investigated (Figure 3). As can be seen in Figure 3, substrate conversion increased rapidly with reaction time and reached its maximum at 120 min, and the regioselectivity towards 7′-OH was >95%. It is interesting that the decrease of 7′-regioselectivity matches the increase of selectivity towards 7′, 6″-diundecenoate with the time elongation. This may be because part of 7′-undecenoate was further esterified to didecenoate with the reaction proceeding. After a reaction time of 150 min, substrate conversion was gradually dropped possibly because of the hydrolysis of 7′-O-undecylenoyl-gastrodin. Therefore, it is significant that the reaction time should be well controlled to gain high conversion and high 7′-regioselectivity in this reaction.

### Effect of organic media

It is well known that the reaction medium could modulate enzyme activity, tailor enzyme selectivity and alter enzyme stability in non-aqueous biocatalysis [20], [21]. Due to the polar nature, glycosides are scarcely soluble in hydrophobic organic solvents, friendly media for the enzyme. Although hydrophilic organic media are good solvents for glycosides, they often strip the essential water off the enzyme molecules and thus inactivate the enzyme [27].

**Figure 4:**
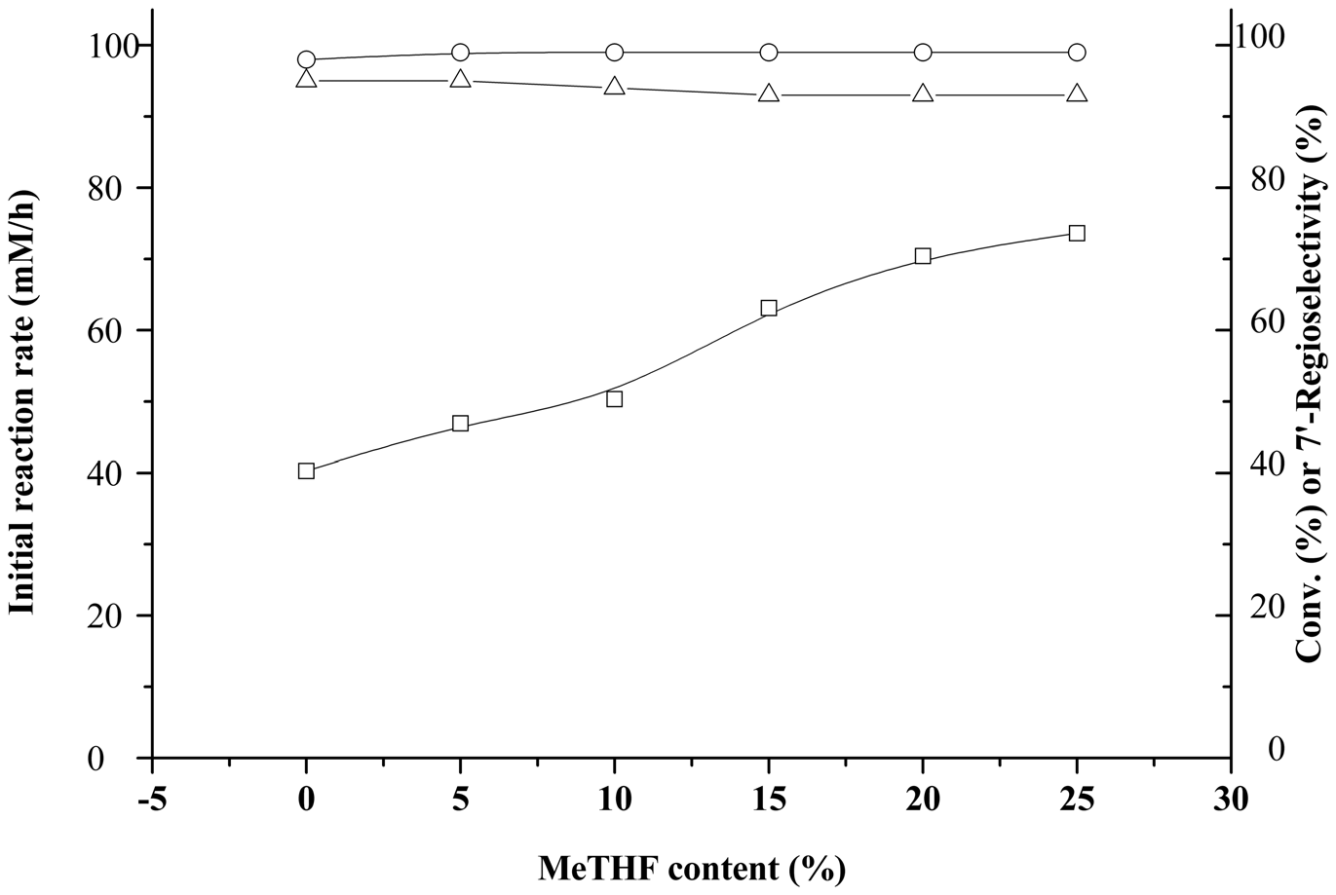
Effect of the MeTHF content on enzymatic undecylenoylation of gastrodin. Reactions conditions: 20 mM gastrodin, 100 mM vinyl undecylenic acid, 15 U lipase PS IM, 2 mL anhydrous organic solvent, 45°C, 200 rpm. Symbols: (□) Initial reaction rate, (○) conversion, (△) 7′-regioselectivity.

As shown in Table 2, no reaction occurred in polar solvents such as DMF and DMSO. This is in agreement with previous results [11], [28]. Good conversions were observed in THF, *t*-Butanol and THF-IL co-solvents containing [C_4_MIM][BF_4_] and [C_4_MIM][PF_6_]. When the volumetric concentration of MeTHF was below 25% in the co-solvent containing MeTHF, the higher the MeTHF content, the higher the initial rate (Figure 4). The reason may be that the presence of an extra methyl group enable MeTHF polarity below THF, so that MeTHF would be more friendly to the lipase [29]. Further increasing MeTHF content beyond 25% reduced substrate solubility less than 20 mM/L. Besides, Table 2 and Figure 4 showed that the reaction media exerted slightly effect on the regioselectivity of this lipase. Generally, THF-MeTHF (3/1, v/v) was consider to be the most suitable reaction media.

**Table 2:** Effect of reaction media on regioselective enzymatic acylation of gastrodin.

Medium	V_0_ (mM/h)	*C* (%)	7'-Ester (%)
t-Butanol	12.8	91	90
THF	40.3	98	95
DMSO	n.d.	n.d.	n.d.
DMF	n.d.	n.d.	n.d.
20%(v/v)MeTHF/THF	70.4	99	93
20%(v/v)[C_4_MIM][BF_4_]/THF	19.3	89	92
20%(v/v)[C_4_MIM][PF_6_]/THF	40.8	98	93

Reaction conditions: 20 mM gastrodin, 100 mM vinyl undecylenic acid, 15 U PS IM, 2 mL anhydrous organic solvent, 45°C, 200 rpm.

n.d.: no detected.

### Operational stability of Lipase PS IM

**Figure 5:**
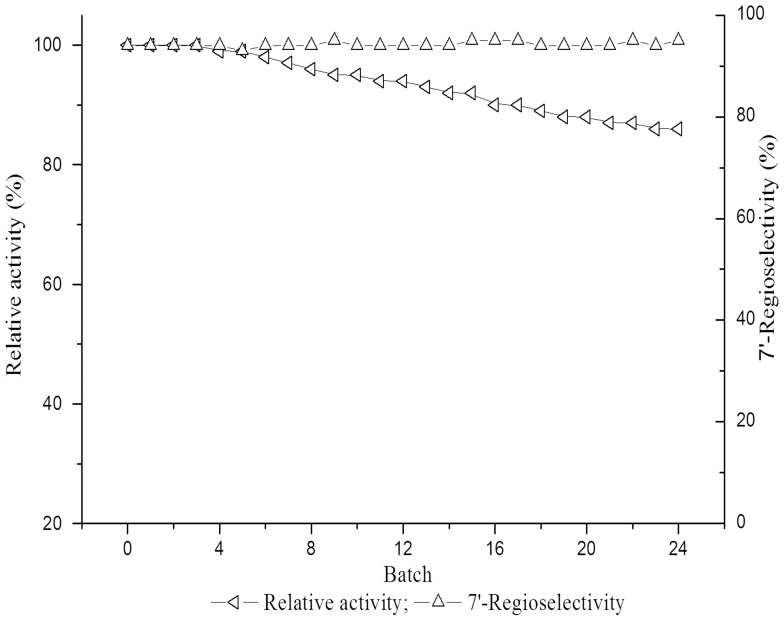
Operational stability of lipase PS IM in 2-methyltetrahydrofuran-containing systems. Reactions conditions: 20 mM gastrodin, 100 mM vinyl undecylenic acid, 15 U lipase PS IM, 2 mL anhydrous THF-Me THF (3/1, v/v), 45°C, 200 rpm.

In order to better examine the potential of recycling lipase PS IM for regioselective acylation of polyhydroxy compounds, its operational stability was investigated. As depicted in Figure 5, lipase PS IM dsplayed the higher operational stability with 14% loss in activity after 24 cycles of the reaction, which highlighted the cost-effectiveness of the enzyme.

As clearly shown by the above study, the enzymatic regioselective preparation of polyhydroxy compound gastrodin is highlighted by the use of lipase PS IM and MeTHF, which provided a mild, efficient, simple and low-cost ‘‘green chemistry’’ methodology for this synthesis process.

## Conclusions

The procedure described here represents a facile and green enzymatic approach for the production of novel gastrodin derivatives with potential pharmacological activities. The activity of lipase PS IM could be markedly enhanced in THF-Me THF (3/1, v/v) than in THF (73.6 vs. 40.3 mM). Using this strategy, efficiently enzymatic acylation of gastrodin was achieved, giving substrate conversion and 7′-regioselectivity exceeding 99% and 93%, respectively. This finding further highlights the versatility of lipases and the potential of “solvent engineering” for modulating enzymatic reactions.
